# Intra-arterial Administration of Human Umbilical Cord Blood Derived Cells Inversed Learning Asymmetry Resulting From Focal Brain Injury in Rat

**DOI:** 10.3389/fneur.2019.00786

**Published:** 2019-08-13

**Authors:** Elzbieta Gornicka-Pawlak, Miroslaw Janowski, Aleksandra Habich, Anna Jablonska, Joanna Sypecka, Barbara Lukomska

**Affiliations:** NeuroRepair Department, Mossakowski Medical Research Center, Polish Academy of Sciences, Warsaw, Poland

**Keywords:** focal brain injury, human umbilical cord blood cells, intra-arterial infusion, functional recovery, asymmetry

## Abstract

**Background:** Focal brain injury is a leading cause of serious disability significantly worsening patients' quality of life. Such damage disrupts the existing circuits, leads to motor, and cognitive impairments as well as results in a functional asymmetry. To date, there is still no therapy to effectively restore the lost functions. We examined the effectiveness of human umbilical cord blood (HUCB)-derived cells after their intra-arterial infusion following focal stroke-like brain damage.

**Methods:** The model of stroke was performed using ouabain stereotactic injection into the right dorsolateral striatum in rats. Two days following the brain injury 10^7^ cells were infused into the right carotid artery. The experimental animals were placed into enriched environment housing conditions to enhance the recovery process. Behavioral testing was performed using a battery of tasks visualizing motor as well as cognitive deficits for 30 days following brain injury. We assessed animal asymmetry while they were moving forward at time of testing in different tasks.

**Results:** We found that intra-arterial infusion of HUCB-derived cells inversed lateralized performance resulting from the focal brain injury at the early stage of T-maze habit learning task training. The inversion was independent from the level of neural commitment of infused cells. The learning asymmetry inversion was observed only under specific circumstances created by the applied task design. We did not found such inversion in walking beam task, vibrissae elicited forelimb placing, the first exploration of open field, T-maze switching task as well as apomorphine induced rotations. Both the asymmetry induced by the focal brain injury and its inversion resulting from cell infusion decreased along the training. The inversion of learning asymmetry was also independent on the range of the brain damage.

**Conclusions:** Intra-arterial infusion of HUCB-derived cells inversed lateralized performance of learning task resulting from focal brain damage. The inversion was not visible in any other of the used motor as well as cognitive tests. The observed behavioral effect of cell infusion was also not related to the range of the brain damage. Our findings contribute to describing the effects of systemic treatment with the HUCB-derived cells on functional recovery following focal brain injury.

## Introduction

The focal brain injury resulting from vascular dysfunction is a leading cause of serious and long-term disabilities of patients (1). To date, there is no efficient therapy to repair damaged tissue as well as to restore the impaired functions (2). Natural endogenous mechanisms triggered to recover lost functions or to develop compensation are unfortunately limited, although they possess an incredible potential (3). Such state of art encourages scientists and clinicians to assess novel therapeutic approaches aimed to restore the lost functions.

To measure the potential effects of experimental therapy on functional recovery following focal brain injury, it is necessary to induce in experimental animals a unilateral damage to the brain tissue similar to those occurring in patients suffering from stroke (4). To accurately evaluate treatment benefit, the brain lesion should be reproducible, relatively small, and causing functional impairments, which would be easy to measure using behavioral tasks allowing to expose disabilities along observation period. To describe the quality of life after such onset followed by a therapeutic intervention, the designed set of behavioral tests should contain tasks revealing motor deficits as well as cognitive impairments providing information concerning the progress in animal recovery.

Cell therapy seems to be a promising approach to improve functions impaired due to brain injury (5–7). Such therapy could enhance the endogenous healing mechanisms (8), as well as to promote structural repair to obtain healthy brain tissue. The cells destined for therapeutic use should meet criteria of safety and ethical acceptance (9). The human umbilical cord blood seems to be such source of cells with potential for restorative therapies (10, 11). One of the major advantage of this cell reservoir is relatively easy accessibility by puncturing umbilical cord at a time of child birth (12). It was previously shown that mononuclear non-hematopoietic cells (CD34^−^) isolated from human umbilical cord blood conversed into neural progenitors lineage after *in vitro* culture (13, 14). As previously described we examined three different populations of human umbilical cord blood-derived cells differing in their level of neural commitment (15). We hypothesized that neurally-committed cells can be more effective for treatment of brain lesion. We compared the therapeutic effectiveness of: (1) freshly isolated human umbilical cord blood mononuclear cells deprived of CD34^+^ population (HUCB-MNC CD34^−^), (D-0); (2) HUCB-MNC CD34^−^ cultured for 3 days in media stimulating neural differentiation (D-3); (3) human umbilical cord blood derived neural stem cell line (NSC) established in our laboratory. The main idea concerning routes of cell delivery was to administer them by infusion into artery supplying blood to the damaged area of the brain. It would potentially maximize the amount of cells reaching the injured tissue. There is a rapidly growing interest in intra-arterial route of stem cell delivery to the CNS. The safety of intra-arterial injections has been solved through adjustment of cell dose and velocity of infusion (16, 17). Real-time MRI allows to make the procedure precise and predictable, what is of utmost importance for translation to clinical setting (18). Moreover, the expression of adhesion molecules is capable to further enhance homing of intra-arterially delivered stem cells (19, 20).

To induce a focal brain injury we used a stereotactic injection of ouabain (OUA) into the rat's right dorsolateral striatum (21). Ouabain is known to trigger the cell death via mechanism similar to those occurring in the absence of an energy supply, observed after an ischemia or a hemorrhage (22). Briefly, the ouabain blocks the Na^+^/K^+^ ATP-ase pump, which was shown ceasing to function after interruption of glucose and oxygen delivery. This leads to membrane depolarization and following molecular events. Such action triggers development of lesion which might be considered as a model of stroke free of vascular dysfunction component. While, in this model brain injury is not caused by the vascular blockade, it has several advantages. The stereotactic surgery is less invasive and much faster than obtaining access to carotid or cerebral vessels, therefore the procedure itself is not producing that much systemic inflammation, which can be a confounding factor in traditional stroke models. Moreover, ouabain model of stroke leaves carotid vessels intact, which facilitates surgery for intra-arterial cell infusion, while the use of carotid vessels for stroke induction complicates their subsequent use for cell infusion a couple days later due to many local, post-operative changes. The size of the stroke can also be easily controlled by the amount of injected ouabain, and the lesion size selected by us was devoid of any mortality. The dorsolateral striatum was chosen as a structure to induce stroke-like damage, since it was shown to be involved in a motor behavior (23), as well as in cognitive processes (24–26). In our model the ouabain injection into dorsolateral striatum was shown to produce significant motor deficits in walking beam task and changed patterns of exploratory behavior in the open field (27). This focal brain injury also caused the cognitive impairments i.e., habit learning and cognitive flexibility deficits (28). To maximize the effectiveness of cell treatment we used the large groups of experimental animals housed in enriched environment conditions. The applied design enables social interactions as well as the opportunity to explore and to exercise the impairments resulting from the brain injury (29). Providing the opportunities of various activities to experimental animals would increase the amount of functional connections in a regenerating tissue and in this way to improve the effectiveness of cell therapy.

Focal brain injury is inseparably related to unilateral damage of the existing, functional circuits, and it results in animal disabilities and lateralization. These symptoms are diversified depending on severity and exact location of the brain damage as well as specific demands in different situations. Experimental therapy is desired to repair them. In the presented study, we evaluated the experimental rat's asymmetry while they were moving forward under various circumstances to quantify and compare effects of cell infusion following focal brain injury.

## Methods

### Animals

Male Wistar rats weighing around 250 g were used for experiments. Animals were kept in enriched environment housing conditions which was introduced just after receiving rats from animal house, 4 days before brain injury, as previously described in detail (15, 27, 28). Enriched environment cages (70 × 41 × 56 cm) contained beams, platforms, ladders etc. Cage equipment was rearranged twice a week. Rats were housed in large groups (7–8 animals per enriched environment cage), 12/12 h light/dark cycle, *ad libidum* feeding with standard feed and tap, filtered water. For the purpose of immunosuppression, Cyclosporine A (Novartis) in dose of 1 mg/kg b.w. daily were administered i.p. to rats throughout the entire experiment. All of the animal handling and experimental procedures were approved by IV Local Ethics Committee on Animal Care and Use (Ministry of Science and Higher Education, Warsaw, Poland).

### Cell Preparation

The isolation and preparation of cell populations derived from the human umbilical cord blood as well as their quantity was maintained according to the protocol described previously (15). Briefly, after delivering the placenta, cord blood was collected by puncturing umbilical cord veins. The mononuclear CD34^−^ cells were obtained using Ficoll separation. HUCB MNC CD34^−^ cells were washed (D-0) or cultured for 3 days in serum free medium (DMEM/F-12+B27+EGF+bFGF, 10^7^ cells/1 ml) (D-3). Then the cells were resuspended in FBS/DMSO and stored in liquid nitrogen for future purposes. Prior to transplantation the cells were quickly thawed at 37°C, washed and resuspended in PBS (10^7^cells/0.5 ml). Cells viability was confirmed by staining with TrypanBlue dye at time of manual cell counting it was no lower than 80%.

Human umbilical cord blood derived neural stem cell (NSC) line (13) is being continuously cultured in our laboratory in low serum medium: DMEM/F12 (Gibco) + ITS (1:100, Gibco) + FBS (2%, Gibco) + AAS (Gibco). Twenty-four hours prior to infusion, the cells were placed in serum free medium (Neurobasal, Gibco). For administration 10^7^ cells were washed and resuspended in 0.5 ml of PBS.

### Surgery

#### Focal Brain Injury

**Figure 1:**
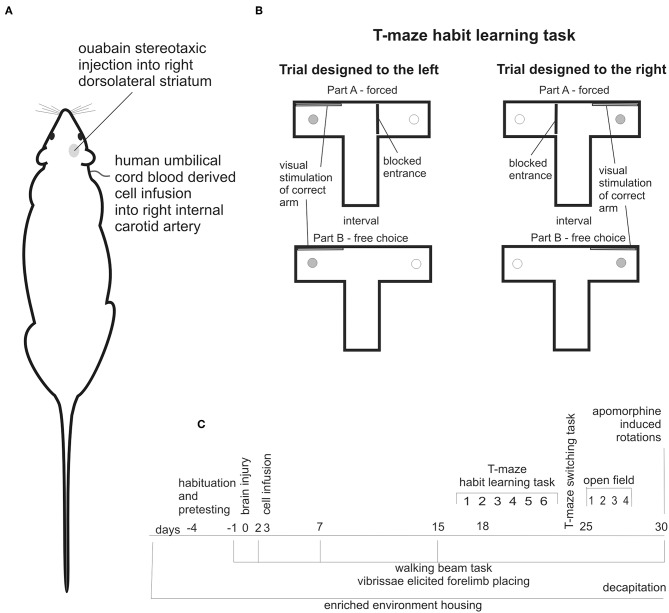
Experimental design: stereotaxic brain injury by ouabain injection into right dorsolateral striatum followed by cell infusion into right internal carotid artery **(A)**, schema of T-maze habit learning task trials designed to the left and to the right **(B)**, schedule of behavioral testing, and housing conditions of all experimental animals along observation period **(C)**.

The stereotaxic ouabain injection into the right dorsolateral striatum (Figure 1A) was made like previously described (15, 27, 28). Briefly, 1.5 μl 5 nmol ouabain (OUA) has been injected with coordinates A: 0.5, L: 3.8, V: 4.7 at a speed 1 μl/min.

#### Cell Infusion

Cell infusions were made into right internal carotid artery (ICA) (Figure 1A). In supine position of experimental animal the skin incision has been made in cervical area followed by the dissection of carotid arteries and after ligation of external carotid artery (ECA), the ICA was punctured and either 10^7^ cells suspended in 0.5 ml of PBS or 0.5 ml PBS was injected over a period of 3–5 min. The needle has been withdrawn and after careful hemostasis the ICA has been left patent.

### Behavioral Testing

#### Walking Beam Task

The rats were trained, like previously described (27) to walk along a narrow wooden beam (14 mm wide, 800 mm long, elevated 50 cm above the cushioned floor) to reach other 3 experimental animals being in the standard cage located at the end of the beam. To evaluate quality of using both the left and right limbs simultaneously while walking along beam, a mirror was set up a side of the apparatus. Each experimental rat performing the task was video recorded from the left side together with its right side mirror view. The rats were tested before brain injury and then 2, 7, 15, and 30 days following lesion. The quality of walking was evaluated quantitatively in developed previously 8-points scale (27). Briefly: unable to move - score 0, able to shift with severe difficulties - score 1, traversing whole beam length, both contralateral paws placed aside of the beam with deep sleeps - score 2, deep sleeps of the left hind limb, placing feet aside of the beam much lower than top of the beam - score 3, slight sleeps of the left hind limb, placing aside close to the upper edge - score 4, irregular consistently asymmetrical left hind limb stepping that is shorter steps, or another shape of moving limb or placing left hind paw with fingers aside of the top of the beam in compare with right hind limb moving and placing straightly - score 5, irregular, symmetric stepping, both left and right limbs incidentally shorter steps, or fingers placed aside of the top of the beam - score 6, perfect walk completely symmetric - score 7. Rats were evaluated from video records by experimenter blind for treatment.

#### Vibrissae Elicited Forelimb Placing

Same side vibrissae elicited forelimb placing test was performed accordingly (30). Briefly, Each rat was handled to feel relaxed. Then the animal was held by hind limbs and one of forelimbs were constrained. Only a single forelimb was free to move. The experimenter moved each rat toward the edge of a table to touch it with its vibrissae same side to unconstrained fore limb. The rat moved extending free forelimb to defend its head. The animals were tested for both left and right forelimbs, using accordingly both left and right vibrissae, before, 2, 7, 15, and 30 days following brain injury. The scores were noted as able or unable to move free forelimb to place at the incoming edge.

#### T-Maze Habit Learning Task

The T-maze habit learning task was performed like previously described (28). Briefly, the T-maze was made of black wood. Each arm contained a 1 cm deep food well at the end, so a rat was unable to see the cookie from the choice point. Before the surgery, the rats were habituated to the T-maze and food reward. The cognitive testing began 16 days following brain injury. At this time the rats did not show any symptoms like weakness, strong need to sleep, or lack of interest in surrounding environment, which would worsen cognitive performance independently on impairments resulting from the brain damage. We did not use any food restriction. On the 16th day following brain injury, the rats performed six trials of the T-maze habit learning task to familiarize them with the test protocol. Regular training begun on the 17th day following the brain lesion. At time of testing rats were kept in waiting boxes close to the apparatus. Each trial consisted of two parts: forced and free choice (Figure 1B). In the forced part, one arm of the maze was blocked at its entry with guillotine door and the only one bitted with food reward was available for the rat released from starting compartment of the maze. Next, after consuming the cookie rat was moved outside the maze for interatrial interval, food well was refilled with piece of cookie, guillotine door was removed and the free choice part was conducted, in which the released rat could enter both arms. After consuming the cookie rat was moved back to the waiting box. The correct arm in the free choice part was the same as in the forced part. In addition, the correct arm in both parts was indicated by a visual clue - a white tablet on the wall. Rats were trained one by one. The daily sessions consisted of 10 trials: 5 designed to the left and 5 designed to the right arranged randomly with unpredictable order (Figure 1B). No more than two consecutive trials in the same direction were allowed. The T-maze habit learning task training was performed for 6 days. The number of correct responses made by each experimental animal during daily training that is turn first of all into correct arm in free choice part was noted as a positive result. In case of an incorrect choice, that is firs entry into unmarked, unvisited during forced part arm, the rat was allowed to find and consume the food reward in the correct arm and the result was noted as negative score. Single rat was handled to avoid too much scare as well as too much arousal prior to each trial.

To evaluate the unilateral lesion-induced lateralization, we analyzed separately the number of correct responses when trials were designed to the left and to the right (Figure 1B). To quantitatively describe T-maze habit learning task lateralization we expressed the number of correct responses made when trial was designed to the left as % of total correct responses.

### T-Maze Switching Task

Just after completion of the habit-learning training, the rats were tested on the switching task using the same maze. The T-maze switching task consisted only of free choice trials in which visual stimulation was the only relevant clue. The rats needed to switch not to use information concerning which arm was previously visited. We used 20 trials performed along single day, an equal number of trials designed to the left and to the right and no more than two consecutive turns in the same direction. Similarly as in the T-maze habit learning task, if rat entered the incorrect arm, it was left in the T-maze until reward was found. The results and lateralization were scored as in the preceding task.

#### Open Field Test

The open field test was performed 25 days following the surgery. Each rat was placed in the center of the large, circular, black arena (1 m diameter surrounded by 30 cm height wall). On the first day individual rats sessions lasted 30 min. On the succeeding 3 days sessions 10 min were performed. The sessions were video recorded and the body center point of the freely moving rat was captured by the EthoVision video tracking system (Noldus). Only first 10 min of first session were used here. To analyze the raw xy coordinates of the rat body center SEE Workshop was applied (31). To evaluate tendency of each rat turning, a parameter Median Curvature of Progression Segments was multiplied by −1 and named locomotor curvature.

#### Apomorphine Induced Rotations

Thirty days following the surgery the rats were injected with apomorphine (0.75 mg/kg b.w., s.c.) and placed in individual circular arenas. The animals were video recorded for 60 min. The number and direction of rotations were scored from video recording by experimenter blind for treatment.

### Histological Analysis of Rat Brain Tissue

Histological analysis was made like we previously descried (15). Briefly, 30 days following brain injury the rats were anesthetized with halothane and decapitated. The brains were quickly removed and immediately frozen in powdered dry ice and stored at −70°C. Every 5th of 20 μm thick coronal sections was stained with a standard hematoxylin and eosin (H&E) method. For quantitative measure of lesion volume images of every fifth of stained sections, visualized in the Discovery v. 1.2 (Zeiss) stereoscope microscope, were analyzed using Corel PHOTOPAINT software. Then values of cavity extent of preceding sections of each rat's brain were multiplied by corresponding brain block thickness (which was 500 μm) and summarized. For analysis of exact location and extend of the damaged tissue we chose the brain area ranging to end of striatum in rostral direction and at hippocampus level in caudal direction. Within this part of the brain the 5 coronal, subsequent planes were selected. Then the corresponding images of H&E stained sections of the brain of each rat were collected and arranged one by one. In next step, the printed representative juxtapositions of the injured brains of all animals included in one experimental group were arranged side by side. Then the minimal and maximal lesion extension were manually overdrawn on brain structures schemas. For the vector graphics Corel DRAW software were used.

### Data Analysis and Statistics

Statistical analyses were performed for all experimental animals, which completed every designed procedures (Figure 1C), i.e., performed walking beam task before brain injury without visible asymmetry, successfully underwent surgery, showed motor impairments in walking beam task 2 days following brain injury, obtained cell intra-arterial infusion, were tested in all behavioral tasks without technical difficulties and their brains were stained for histological analysis. We did not reject any of experimental animals which met above criteria.

To evaluate the data, a non-parametric statistical analysis was applied. To find if one of compared groups was significantly different from other ones the Friedman ANOVA analyses were used. To estimate which of experimental groups is significantly different form other ones the Mann-Whitney *U*-test for non-matched pairs was calculated. Data on all graphs are medians ±25 and 75%.

## Results

### T-Maze Habit Learning Task

**Figure 2:**
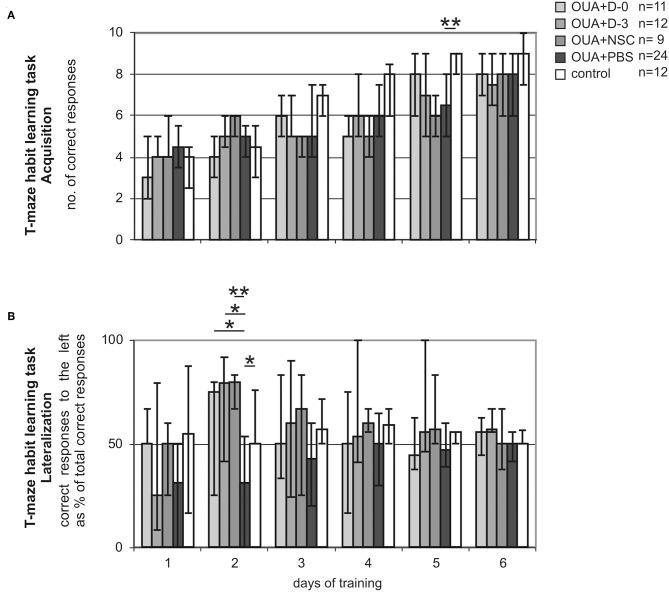
T-maze habit learning task training. Acquisition **(A)** expressed as the number of correct responses made by rats treated either with human umbilical cord blood derived cells being at different stages of neural commitment or PBS and control ones. Lateralization **(B)** expressed as the amount of correct responses made by experimental animals when trial was designed to the left conveyed as a % of all correct responses along consecutive days of training. Line marked on the graph at value of 50% points out the direction of learning asymmetry (median ± 25 and 75%; *p < 0.05, **p < 0.01 in Whitney-Mann test for non-matched pairs).

We assessed T-maze habit learning task training of experimental animals. Acquisition of the task was juxtaposed with its lateralization to visualize animal asymmetry at different stages of learning process. As we previously described (15, 28) OUA injection into the right dorsolateral striatum impaired acquisition of T-maze habit learning task (Figure 2A). Injured rats treated with PBS made significantly less correct responses at 5th day of training than control ones (*p* < 0.01). OUA animals treated with either D-0 or D-3 cells showed tendency to improve impaired by the brain injury task acquisition, but this difference did not reach statistical significance.

T-maze habit learning task Lateralization, that is amount of correct responses made by experimental rats when trial was designed to the left expressed as percent of total correct responses was significantly shifted by focal brain injury (Figure 2B). OUA injection into the right dorsolateral striatum resulted in performing significantly less correct responses when trial was designed to the left in compare with those designed to the right at the early stage of acquisition that is second day of training. Treatment with all cells populations inversed the direction of task lateralization. D-0. D-3 (*p* < 0.05) and NSC (*p* < 0.01) cell treated groups of rats showed significantly more correct responses when trial was designed to the left then to the right in comparison with OUA+PBS group at second day of training. Control animals performed the task at comparable amount of correct responses made when trial was designed to the left and to the right through all training.

T-maze habit learning task Lateralization was the most inclined at 2nd day of training in both OUA+PBS and mirror view inversed in cell treated groups. Lateralized task performance at the early stage of training improved with time along consecutive sessions in all OUA groups. At the last day of training that is 6th, all experimental groups of rats performed the task at comparable amount of correct responses made when trial was designed to the left and to the right.

### Juxtapositions of Rat Asymmetries Visible in Various Behavioral Tasks

**Figure 3:**
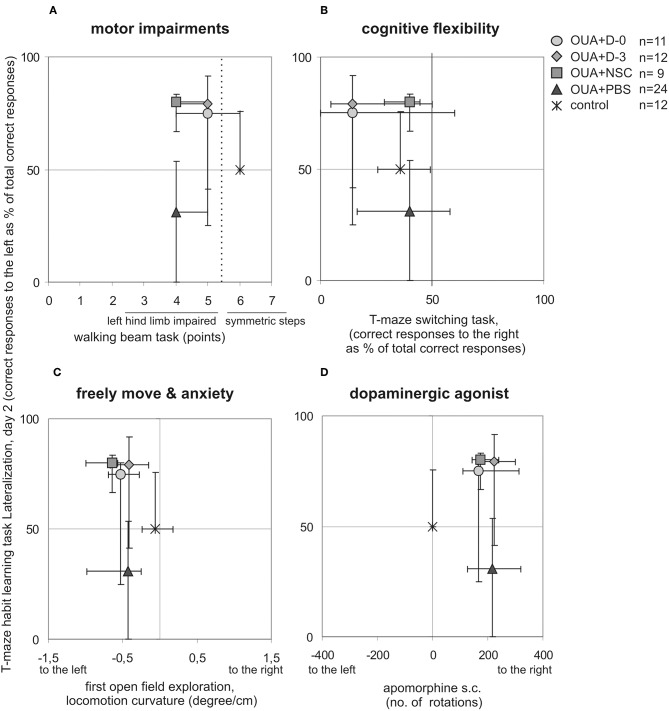
Comparison of experimental animals asymmetries visible in different behavioral tasks. T-maze habit learning task lateralization at 2nd day of training was juxtaposed with: motor impairments of left hindlimb in walking beam task 15 days following brain injury **(A)**, cognitive flexibility in T-maze switching task **(B)**, turning tendency when rat was freely moving under anxiety in first open field task session **(C)**, number and direction of rotations induced by dopaminergic agonist apomorphine s.c. injection **(D)**, (median ± 25 and 75%, lines marked on the graphs point out direction of presented asymmetries).

T-maze habit learning task Lateralization at 2nd day of training was juxtaposed with asymmetries visible in other behavioral tasks performed in single animals to describe functional impairments resulting from focal brain injury and potential recovery induced by cell infusion (Figure 3). Learning asymmetry was compared with: motor deficits, that is level of left hind limb impairments visible in walking beam test performed 15 days following brain injury; cognitive flexibility lateralization measured in T-maze switching task; turning tendency in the open field first 10 min exploration when rat was freely moving in novel empty arena and number as well as direction of rotations induced by non-selective dopaminergic agonist apomorphine s.c. injection. We also compared T-maze habit learning task Lateralization at 2nd day of training with vibrissae elicited left forelimb placing permanent deficit across all observation period. None of juxtaposed asymmetries of experimental animals under various circumstances were inversed by cell infusion like it was shown for T-maze habit learning task Lateralization at 2nd day of training.

**Figure 4:**
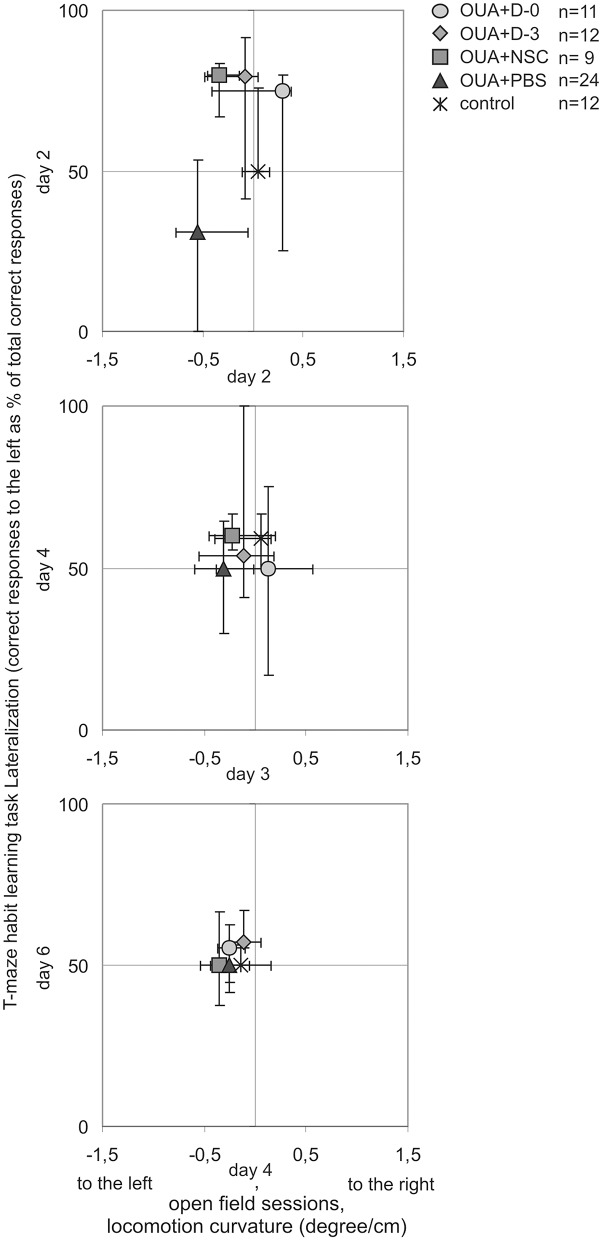
Comparison of asymmetries by experimental animals along T-maze habit learning task training and consecutive open field sessions. Lateralization of cognitive task performance was juxtaposed with turning tendency of freely moving rats in corresponding subsequent days of testing (median ± 25 and 75%).

Lateralization of T-maze habit learning task performance along training was also juxtaposed with turning tendency of freely moving single rats during consecutive open field 10 min sessions (Figure 4). In contrast to learning asymmetry at 2nd day of training, when all of examined treatments with cell populations induced inversion, at 2nd day of open field testing only OUA+D-0 rats showed opposite direction of median locomotor curvature, that is to the right (which however was statistically insignificant). Both compared asymmetries induced by focal brain injury and modified by cell treatment decreased with preceding sessions. Turning tendency at the end of open field testing remained a little to the left in contrast with T-maze habit learning task performance, reaching in the last training session comparable amount of correct responses to the left and to the right in all experimental groups.

### Learning Asymmetry and Brain Injury Range

**Figure 5:**
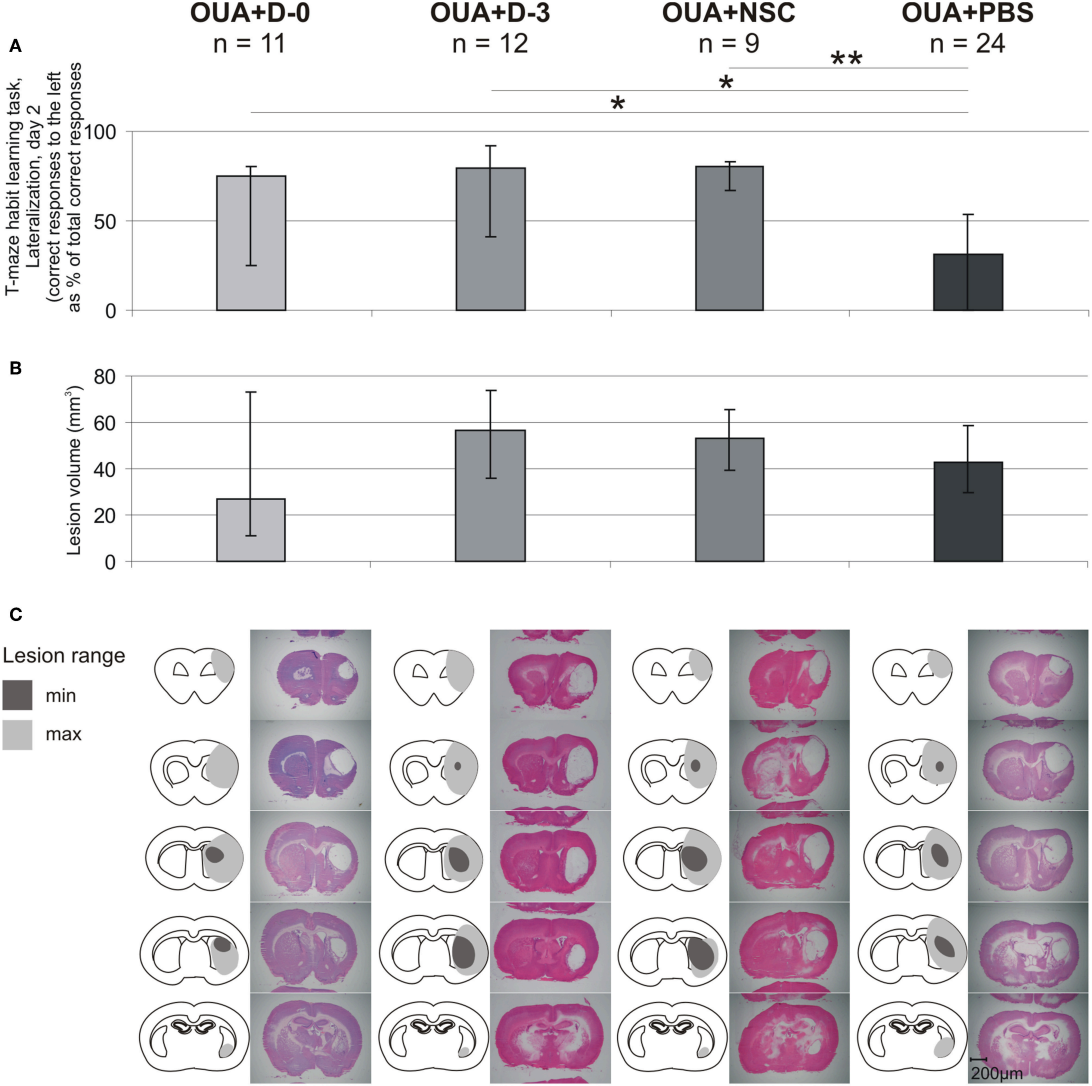
Comparison of learning asymmetries and brain injury range. T-maze habit learning task Lateralization at 2nd day of training **(A)**, lesion volume **(B)**, lesion extent and representative images of H&E stained coronal brain slices **(C)** for each of experimental groups (median ± 25 and 75%; *p < 0.05, **p < 0.01 in Whitney-Mann test for non-matched pairs).

T-maze habit learning task Lateralization at 2nd day of training was also compared with range of brain damage (Figure 5). In contrast with statistically significant difference between OUA+PBS rats and all of three cell treated groups, in the level of learning asymmetry there were no significant differences between experimental groups in both lesion volume as well as cavity location and extension.

## Discussion

We found that the direction of considerable learning asymmetry resulting from focal brain injury was significantly inversed following intra-arterial infusion by each of the three examined populations of the human umbilical cord blood derived cells. Such common functional effect of applied therapy was observed although cell fractions differed in level of their neural commitment. Both the lateralization of T-maze habit learning task performance resulting from focal brain injury and the inversion of its direction following the cell infusion were observed only under specific circumstances, that is at the early stage of the task training. Such an inversion resulting from the cell infusion was not observed in any of asymmetric motor impairments that is left hind limb disabilities while walking along balance beam and vibrissae elicited left forelimb placing deficit. The inversion of asymmetry induced by unilateral brain injury followed by experimental treatment was also not observed in another cognitive task measuring animals behavioral flexibility and possibility to adapt to novel rules in the same environment. The asymmetry inversion resulting from the applied therapy in injured animals also did not concern the direction of forward movement of freely moving rats under anxiety as well as the direction of rotations induced by dopaminergic agonist apomorphine administration. Insignificant tendency to inverse the median direction of forward movement of freely moving rats at the early stage of open field testing sequence was observed only in OUA+D-0 rats. This is in agreement with our previous results that D-0 freshly isolated cells were the most effective in restoration of impaired function and decreasing lesion volume (15). Both the learning asymmetry and the freely move curvature improved along T-maze habit learning task training and open field consecutive testing.

In the present study we applied criteria of the experimental animal selection analysis. That is all included rats underwent testing in all of the considered behavioral tasks and subsequent histological analysis. Such approach seems to decrease experimental bias. It should be taken into consideration that each of the uninjured rat possess its natural asymmetry while solving T-maze task (32). Therefore, the individual animal asymmetry was overlapped by the individual vulnerability for cytotoxic brain injury which corresponded with the variable lesion volume observed in our study. Rats also differ in their natural capacity to restore or to compensate the impaired functions following brain damage, which corresponded with a lack of the correlation between the lesion volume and the behavioral tasks scores in our experiment. Such diversity was also influenced by the individual animal reactivity to xenogenic cell infusion. An evaluation of the cohort of experimental animals meeting selection criteria would provide possibility to compare more accurately the asymmetry of rats behaving under various circumstances.

To promote recovery of healthy brain tissue we used enriched environment housing conditions (29). Such housing, due to natural need of animals to explore surroundings, enables motor and cognitive exercising in three dimensional complex space equipped with various beams, ladders, platforms etc. differing in width, shape, and their position in space. Abundant social interactions additionally ensure establishment of proper functional recovery. Rearrangement of equipment within the cages during the experiment promoted rats abilities to find them serf in partially novel environment. Altogether, the above processes would facilitate a functional restoration and improve the cell infusion effectiveness by reducing focal brain injury induced asymmetry occurring under various circumstances. This is due to experimental rats were freely moving in enriched environment home cage and all activities, movements, paying attention or noting that something appeared were possible to the left as well as to the right side.

### T-Maze Habit Learning Task Training

T-maze habit learning task employed “win-stay” paradigm rules in T-maze, which is rat needed to choose the arm of maze visited a while ago (28). On one hand the task requires that rat has to overcome natural preference to choose novel, previously unvisited environment. On the other hand if rat is anxious it prefer familiar, previously visited environment. Along 6 days of T-maze habit learning task training, rats at first were scared but explore the maze, learned that food reward was possible to find in one of food wells located at the end of each maze arms. Then the animals started to find out that the task was required from them. It occurred approximately on the second day of training. Then rats learned the task by the method of trials and mistakes. It seems to be raised up by natural drive to find the reward as result of first choice. Injured rats presented difficulties to acquire task design on about 5th day of training.

The most inclined task performance lateralization observed at second day of training and its inversion by the cell infusion would reflect how much the set of emotions at this stage of training influenced decision making at the T-maze choice point. Accordingly, such set of processes would facilitate in injured animals turning into ipsilateral side, while as the effect of cell therapy turning into contralateral side were easier for experimental rats. Another explanation is that more correct responses when trial was designed to the left would reflect the positive therapy effect on social preference. This is due to experimental oversight, that means waiting boxes in which all experimental animals were placed at time of daily task training were located at left side of the T-maze. Such distribution of experimental equipment in space would influence rat's choice inside of enclosed training apparatus. It actually means that cell infusion with each of three examined populations would increase the choosing by experimental rat under specific circumstances T maze arm located closer to other animals, promoting this way preferences for being in company.

### Experimental Animals Asymmetries Following Focal Brain Injury and Human Umbilical Cord Blood Derived Cells Systemic Treatment

The effects of systemic treatment of human umbilical cord blood derived cells on functional recovery following focal brain injury were studied before. Vendrame and coworkers assessed effectiveness of human umbilical cord blood derived cells doses (33). They showed, that direction as well as quantity of spontaneous rotations may significantly differ between 2 and 4 weeks following surgery depending on assessed cell dose.

The results of systemic treatment of human umbilical cord blood derived cells on cognitive impairments were studied in passive avoidance task (34), and water maze task (35). The authors showed cell treatment effects on learning deficits resulting from focal brain injury. Because of both tasks were designed not to be affected by eventual rat asymmetry resulting from focal brain injury, the issue of turning direction at time of tasks performance were not shown.

Effectiveness of systemic treatment with administration of the human umbilical cord blood derived cells on recovery of motor functions were also studied (35–37). In all experiments, the authors reported contralateral limbs impairments and tendency to improvement of impaired side of body following applied therapy. This is in agreement with our results concerning motor recovery following cell infusion.

Often used behavioral task was Neurological Severity Score (38–40). Such task allows to evaluate in details rat's reflexes, ability to move, body/muscle quality, possibility to flex. Because of such detailed assessing NSS is an abundant source of information concerning experimental animal asymmetry in various circumstances. The results are usually expressed as one graph describing overall rat recovery making possible to easy follow therapy effects on rats well-being. Such way of presenting shows general improvement, but unfortunately makes impossible to conclude and to compare the amount and the direction of experimental animal single asymmetries.

Our findings seem to contribute to understanding the effects of HUCB-derived cells treatment following focal brain injury, emphasizing the performance asymmetry of complex impaired by the brain lesion cognitive task at time of acquiring it. Knowledge concerning possible therapeutic effects of HUCB-derived cell treatment would to be beneficial in connection with public interest in umbilical cord tissue banking and stem cell transplantation. However, before translating our results into clinics, it should be taken into consideration that we assessed effects of human umbilical cord blood derived cells using animal model of man disease.

There are several limitations in our study. Our previous paper provided more extensive assessment of outcomes including cell survival in the same experimental set up. Here, we have focused on a very time-consuming and meticulous behavioral task, which revealed that therapeutic benefits can be also related to a very fine cognitive tasks, which may actually have a high impact on a human life. However, still we did not study the influence of intra-arterial xenograft delivery on the host immune response as well as we did not assess whole-body biodistribution of transplanted cells.

## Conclusions

Intra-arterial infusion of cells derived from human umbilical cord blood inversed learning asymmetry caused by focal brain injury. The resulting effect of the applied therapy was independent on the infused cell progress in their neural commitment. Inversion of the asymmetry direction was observed only under specific circumstances, that is at the early stage of training in T-maze habit learning task. Cell infusion did not change significantly the direction of unilateral brain injury induced asymmetry visible in motor tests, cognitive flexibility task as well as while rats were freely moving in novel open arena or administered with dopaminergic agonist apomorphine. Insignificant tendency to shift the median direction of forward movement was only observed in rats treated with undifferentiated cells at the early stage of open field tests sequence. The asymmetries decreased along consecutive testing and familiarization of experimental animals with tasks in both T-maze habit learning task and open field test. Inversion of learning asymmetry due to the applied therapy was also independent on range of the brain damage.

## Data Availability

The datasets generated for this study are available on request to the corresponding author.

## Ethics Statement

All of the animal handling and experimental procedures were approved by IV Local Ethics Committee on Animal Care and Use (Ministry of Science and Higher Education, Warsaw, Poland).

## Author Contributions

EG-P carried out animals behavioral testing and histological analysis. MJ performed surgeries. AH carried out cell preparation for infusion. AJ participated in surgery and cell preparation. JS promoted manuscript drafting and English proofing. BL supervised the project.

### Conflict of Interest Statement

The authors declare that the research was conducted in the absence of any commercial or financial relationships that could be construed as a potential conflict of interest.
